# Blood–brain barrier leakage in relation to white matter hyperintensity volume and cognition in small vessel disease and normal aging

**DOI:** 10.1007/s11682-018-9855-7

**Published:** 2018-03-23

**Authors:** C. Eleana Zhang, Sau May Wong, Renske Uiterwijk, Walter H. Backes, Jacobus F. A. Jansen, Cécile R. L. P. N. Jeukens, Robert J. van Oostenbrugge, Julie Staals

**Affiliations:** 1 Department of Neurology, Maastricht University Medical Centre, P. Debyelaan 25, 6202 AZ Maastricht, the Netherlands; 2 Cardiovascular Research Institute Maastricht (CARIM), Maastricht, The Netherlands; 3 School for Mental Health and Neuroscience (MHeNs), Maastricht, The Netherlands; 4 Department of Radiology & Nuclear Medicine, Maastricht University Medical Centre, Maastricht, The Netherlands

**Keywords:** Blood–brain barrier, Cerebral small vessel disease, Cognition, White matter hyperintensities, MRI

## Abstract

Blood–brain barrier (BBB) leakage increases with age and is involved in the pathophysiology of cerebral small vessel disease (cSVD). We examined the relationship between BBB leakage and white matter hyperintensity (WMH) volume and cognition, in cSVD patients and healthy controls. Seventy-seven patients with clinically overt cSVD and thirty-nine age matched healthy controls underwent dynamic contract-enhanced and structural brain MRI and neuropsychological assessment. We quantified BBB leakage volume and rate in normal appearing white matter (NAWM), WMH and cortical grey matter (CGM). Larger leakage volume and lower leakage rate in WMH were associated with larger WMH volume in cSVD but not in controls. Higher leakage rate in NAWM was associated with lower scores on executive function and information processing speed in healthy controls, whereas no relation with cognition was found in cSVD patients. Our findings support the involvement of BBB leakage in cSVD and aging. They also suggest that the mechanism of cognitive dysfunction in cSVD is more complex and multifactorial in cSVD compared with normal aging.

**Electronic supplementary material:**

The online version of this article (10.1007/s11682-018-9855-7) contains supplementary material, which is available to authorized users.

## Introduction

Blood–brain barrier (BBB) permeability increases with age and dysfunction of the BBB is thought to be an important pathophysiological mechanism in cerebral small vessel disease (cSVD) (Skoog et al. 1998; Wardlaw et al. 2003). cSVD is an age and vascular risk factors related disorder of the small vessels of the brain, and can cause debilitating disorders including vascular cognitive impairment (VCI) and lacunar stroke (Pantoni 2010). Several dynamic contrast-enhanced (DCE) MRI studies observed higher BBB leakage in patients with lacunar stroke and patients with VCI, compared with controls, either in terms of leakage rate or proportion of brain volume showing BBB leakage (Taheri et al. 2011; Topakian et al. 2010; Wardlaw et al. 2009; Zhang et al. 2017).

Macroscopically visible lesions such as white matter hyperintensities (WMH) may occur with increasing age and in the absence of cerebral vascular diseases. However, WMH – especially when they are extensive – are also considered local end-stage phenomena of cSVD and form a surrogate marker of cSVD disease severity. It would be relevant to know whether BBB defects could be tracked as an early marker for cSVD. Although data are limited, higher BBB leakage has been associated with increasing WMH load in healthy older subjects (Farrall and Wardlaw 2009; Skoog et al. 1998). In patients with cSVD, data are even more scarce. No association was found between BBB leakage and WMH volume in patients with VCI (Huisa et al. 2015). In patients with mild stroke, BBB leakage in the NAWM directly surrounding the WMH was higher in patients with the largest WMH load (Wardlaw et al. 2017).

Further, how BBB leakage relates to clinical measures such as cognition is essential for the understanding of the pathophysiology of cSVD. Patients with vascular dementia demonstrated higher BBB leakage compared with healthy controls (Farrall and Wardlaw 2009). Recently, a study in lacunar stroke patients showed no cross-sectional associations between BBB leakage and cognition, but BBB leakage in the WMH was associated with lower cognitive score at one year follow up (Wardlaw et al. 2017). Further data on this matter in cSVD patients are lacking. In normal aging, the relationship between BBB leakage and the level of cognitive function has not yet been studied.

In the present study, we aimed to determine the relationship between BBB leakage and WMH volume, and between BBB leakage and cognition in cSVD and in normal aging controls.

## Material and methods

### Patient population

Patients with clinically overt cSVD were eligible for inclusion in this present study: first-ever lacunar stroke patients or patients with mild vascular cognitive impairment (mVCI) due to presumed cSVD (Zhang et al. 2017). Lacunar stroke patients were recruited from the Stroke Unit of the Maastricht University Medical Centre and Zuyderland Medical Centre, The Netherlands, in the period between April 2013 and December 2014. We defined lacunar stroke as an acute stroke syndrome with a compatible recent small subcortical infarct on clinical brain MRI. In cases in which no lesion was detected on MRI, we used established clinical criteria for lacunar syndrome (Bamford et al. 1987; Wardlaw et al. 2013b). Patients were excluded when they had a potential cardiac embolic source (e.g. atrial fibrillation) or ipsilateral carotid stenosis of ≥ 50%. Lacunar stroke patients underwent the test batteries at least three months post-stroke in order to avoid acute stroke changes. Patients with mVCI due to presumed cSVD were recruited from the outpatient clinic of the Department of Neurology and from the Memory Clinic of the Maastricht University Medical Centre, and Zuyderland Medical Centre. Criteria of mVCI consisted of (1) subjective complaints of cognitive functioning, (2) objective cognitive impairment in at least one cognitive domain at neuropsychological testing, (3) a Clinical Dementia Rating of ≤ 1 and a Mini Mental State Examination score of ≥ 20, and (4) vascular lesions on clinical brain MRI that suggest a link between the cognitive deficit and cSVD (Gorelick et al. 2011): moderate to severe WMH (Fazekas score deep WMH > 1 and/or periventricular WMH > 2), or mild WMH (Fazekas score deep WMH = 1 and/or periventricular WMH = 1–2) with lacunes and/or microbleeds (Fazekas et al. 1987; Zhang et al. 2017).

Furthermore, we included age- and sex- matched healthy controls from the outpatient clinic of the Department of Neurology. One control was included per two cSVD patients. Criteria for healthy controls: no history of overt cerebrovascular diseases and no cognitive impairment. Moreover, none of the participants (patients as well as controls) may have neurodegenerative diseases, multiple sclerosis, epilepsy, systemic inflammatory diseases, alcohol abuse, psychiatric disorders or use of medication that may influence the accuracy of neuropsychological testing, and the presence of a contra-indication for MRI (e.g. pacemaker, claustrophobia, or contrast allergy) (Zhang et al. 2017).

We recorded characteristics including age, sex, educational level and the presence of cardiovascular risk factors including hypertension (history of hypertension and/or use of blood pressure lowering drugs), hypercholesterolemia (history of hypercholesterolemia and/or use of statin), diabetes mellitus (history of diabetes mellitus or use of glucose lowering drugs), smoking (current smoking) and Body Mass Index (BMI: weight divided by the square of length).

The study gained approval of the Medical Ethical Committee of the Maastricht University Medical Centre. All participants gave written informed consent. The study is registered on http://www.trialregister.nl (NTR number NTR3786).

### Magnetic resonance imaging

MRI was performed on a 3.0 T magnetic resonance scanner (Achieva TX, Philips Healthcare, Best, the Netherlands), employing a 32-element head coil suitable for parallel imaging. Structural MRI included a T1-weighted sequence (TR/TI/TE = 8.3/800/3.8 ms; field of view (FOV) 256 × 256 × 160 mm^3^; 1.0 mm^3^ cubic voxel) and T2-weighted FLAIR sequence (TR/TI/TE = 4800/1650/299 ms; FOV 250 × 256 × 180 mm^3^; 1.0 mm cubic voxel). These were used for anatomic reference and detection of WMH, respectively (Zhang et al. 2017).

Dual-time resolution DCE-MRI was composed by two integrated dynamic sequences with different dynamic scan time (DST), the fast and the slow sequence (Zhang et al. 2017). Prior to bolus injection, pre-contrast scans of both sequences were acquired, followed by the fast sequence (DST 3.2 s, TR/TE = 5.6/2.5 ms, FOV: 256 × 200 × 50 mm^3^, voxel size of 2 × 2 × 5 mm, 29 volumes, SENSE = 2) during bolus injection. This fast sequence was followed by the slow sequence (DST 30.5 s, TR/TE = 5.6/2.5 ms, FOV: 256 × 256 × 100 mm^3^, voxel size of 1 × 1 × 2 mm, 45 volumes, SENSE = 2). Contrast agent (Gadobutrol; dose 0.1 mmol/kg body weight) was infused in the antecubital vein at a rate of 3 ml/s using a power injector. To convert the contrast enhanced signal intensities to concentrations in tissue, T1-mapping (Larsson et al. 2009) was performed prior to dynamic contrast-enhanced imaging (Zhang et al. 2017).

### Image processing

Freesurfer (Fischl 2012) was used to segment grey and white matter from the T1-weighted images. WMHs were delineated on the FLAIR image using a semi-automated segmentation tool (de Boer et al. 2009). Infarcts and lacunes were visually identified and excluded from the WMHs. FLAIR and T1-weighted images were spatially co-registered (FSL version 5.0) (Jenkinson et al. 2002) and the following brain regions were selected: normal appearing white matter (NAWM), WMH, and cortical grey matter (CGM). The WMH volume was quantified and normalized to the intracranial volume (Zhang et al. 2017).

### Pharmacokinetic modelling and histogram analysis

Blood signal in the superior sagittal sinus was used for the vascular input function to calculate the concentration in blood plasma (Lavini and Verhoeff 2010). To avoid contamination of inflow artefacts, the concentration in blood plasma was calculated by using a calibration obtained from phantoms with various in vitro contrast agent concentrations. Then, the graphical Patlak model was used to estimate the leakage rate in terms of the transfer constant *K*_i_ (min^− 1^) and the blood plasma volume in terms of the fractional intravascular space *v*_P_ (Patlak and Blasberg 1985).

We determined *K*_i_ and *v*_P_ from the Patlak plot and discerned the concentration time-course of the extravasating contrast agent, i.e. leakage, from the intravascular concentration. *K*_i_ is the leakage rate is determined from the increase of the tissue concentration over time from a pharmacokinetic model. Here, the return of contrast agent to the blood is neglected in the time period of the measurement.

*K*_*i*_ and *v*_P_ were determined for each voxel and a histogram was obtained for the *K*_i_ values. We corrected for noise by subtracting the negative *K*_i_ value from the original *K*_i_ distribution, resulting in a histogram reflecting the detectable leakage rates. Quantitative BBB leakage measures were obtained using this histogram: mean *K*_i_, indicating the magnitude of the leakage rate, and *v*_L_ which was the remaining area under the histogram curve, representing the fractional volume of leaky brain tissue (i.e. the spatial extent of leaky brain tissue) (Zhang et al. 2017).

### Neuropsychological assessment

Extensive neuropsychological testing was performed and covered three main cognitive domains (Zhang et al. 2017). Tests assessing the memory domain included the Rey Auditory Verbal Learning Test (immediate recall, delayed recall, delayed recognition) and the Digit Span Forward. Tests assessing the executive function domain included the Stroop Colour-Word Test interference score (time of part 3 minus mean time of part 1 and 2), Trail Making Test interference score (time of part B minus time of part A), Category (animals and professions) and Letter Fluency, Letter-Number Sequencing and Digit Span Backward. Information processing speed was determined using the Symbol Substitution-Coding, Trail Making Test part A and Stroop Colour-Word Test parts 1 and 2. Z-scores were calculated for each test: the difference between the individual raw score and the sample mean (i.e. the sample mean of the cSVD group for individuals of the cSVD group, and the sample mean of the control group for individuals of the control group) was divided by the sample SD. We calculated the compound domain scores for each participant by averaging the z-scores within each domain, and an overall cognition compound score was calculated taking the average of the three domain compound scores. The Hamilton Anxiety and Depression Scale test (range 0–42) was used to record depression and anxiety symptoms (Zhang et al. 2017).

### Statistical analysis

We examined the relationship between *v*_L_, *K*_i_ in different brain regions (NAMW, WMH and CGM) and normalized WMH volume, first using univariable linear regression analysis and then multivariable linear regression analysis correcting for age and sex. Subsequently, we examined the relationship between *v*_L_ and *K*_i_ in the different brain regions (independent variables) and the cognitive performance in three cognitive domains as continuous variables (dependent variables), using univariable and multivariable linear regression with correction for age, sex, educational level and Hamilton Anxiety and Depression Score. All analyses were performed in cSVD patients and healthy controls. Statistical analysis was performed using commercial software (SPSS 22.0); p < 0.05 was considered statistically significant.

## Results

Eighty patients with cSVD (44 lacunar stroke patients and 36 mVCI patients) and forty healthy controls underwent MR imaging and neuropsychological assessment. Three patients (one with lacunar stroke and two with mVCI) and one control were excluded for data analysis due to imaging complications or imaging artefacts. Characteristics of the seventy-seven patients and thirty-nine controls included for analysis are shown in Table 1. For cognitive scores on all individual tests, see Supplemental data.

**Table 1:** Characteristics of cSVD patients and healthy controls

	cSVD patients	Controls	
	N = 77	N = 39	*p*-value
Age, years (SD)	70 (11)	69 (12)	0.58
Male (%)	46 (60)	23 (59)	0.94
Educational level			0.58
Low	35 (45)	13 (33)	
Average	27 (35)	20 (51)	
High	15 (20)	6 (15)	
Hypertension (%)	49 (64)	18 (46)	0.07
Hypercholesterolemia (%)	50 (65)	13 (33)	0.001
Diabetes (%)	12 (16)	4 (10)	0.43
BMI kg/m^2^ (SD)	26 (4)	27 (3)	0.07
Smoking (%)	19 (25)	3 (8)	0.03
WMH volume (SE)	0.014 (0.002)	0.005 (0.002)	< 0.001
*K*_i_ (10^− 3^ min^− 1^)			
NAWM (SE)	0.97 (0.04)	1.05 (0.05)	0.20
WMH (SE)	0.85 (0.03)	0.87 (0.05)	0.66
CGM (SE)	1.43 (0.05)	1.49 (0.07)	0.45
*v*_L_ (10^− 2^)			
NAWM (SE)	37.4 (2.15)	31.4 (2.91)	0.11
WMH (SE)	45.5 (2.31)	35.2 (3.01)	0.01
CGM (SE)	20.7 (1.56)	14.7 (1.76)	0.01

*WMH volume* WHM volume normalized to intracranial volume, *BMI* body mass index, *K*_i_ leakage rate, *v*_L_ leakage volume, *NAWM* normal appearing white matter, *WMH* white matter hyperintensities

### BBB leakage and WMH volume

In patients with cSVD, higher *v*_L_ and lower *K*_i_ in WMH was significantly associated with a larger WMH volume (Table 2). These associations remained significant after correcting for age and sex. In the NAWM or CGM, *v*_L_ nor *K*_i_ were associated with WMH volume. In the healthy control group, no significant association was found between BBB leakage in any of the regions and WMH volume (Table 2). After additional correcting for smoking, the results did not change substantially.

**Table 2:** Association between WMH volume and *v*_L_ and *K*_i_, for NAWM, WMH and CGM, in cSVD patients and healthy controls, uncorrected and corrected for age and sex

	WMH volume							
	cSVD				Controls			
	Univariable		Multivariable		Univariable		Multivariable	
	β	*p*-value	β	*p*-value	β	*p*-value	β	*p*-value
*v*_L_								
NAWM	0.079	0.49	0.092	0.39	0.009	0.96	-0.002	0.99
WMH	0.268	0.02	0.267	0.01	0.184	0.26	0.123	0.45
CGM	0.154	0.18	0.111	0.30	0.097	0.56	0.039	0.81
*K*_*i*_								
NAWM	-0.157	0.17	-0.174	0.10	0.069	0.68	0.048	0.77
WMH	-0.267	0.02	-0.264	0.01	0.159	0.33	0.065	0.70
CGM	-0.223	0.05	-0.185	0.08	0.115	0.49	0.178	0.28

*v*_*L*_ leakage volume, *K*_i_ leakage rate, *NAWM* normal appearing white matter, *WMH* white matter hyperintensities, *CGM* cortical grey matter

β values represent standardized regression coefficients

### BBB leakage and cognitive function

In cSVD patients, no significant associations were found between *v*_L_ or *K*_i_ and the cognitive domains overall cognition, executive function, information processing speed and memory, in univariable (Table 3) and multivariable analysis correcting for age, sex, educational level and Hamilton Anxiety and Depression Score. In healthy controls, higher *K*_i_ in the NAWM and in the WMH was significantly associated with lower scores in cognitive domains executive function and information processing speed, and higher *K*_i_ in the CGM was significantly associated with lower scores in the cognitive domain memory (Table 4). After correcting for age, sex, educational level and Hamilton Anxiety and Depression Score, the association between *K*_i_ in the NAWM and executive function and information processing speed remained significant (β=-0.280 p = 0.03 and β=-0.261 p = 0.02 respectively).

**Table 3:** Association between cognition and *v*_L_ and *K*_i_, for NAWM, WMH and CGM, in cSVD patients, uncorrected

	Overall		Executive		Speed		Memory	
	β	*p*-value	β	*p*-value	β	*p*-value	β	*p*-value
*v*_L_								
NAWM	0.108	0.35	0.118	0.31	0.074	0.52	0.090	0.44
WMH	0.034	0.77	0.071	0.54	-0.006	0.96	0.029	0.80
CGM	-0,028	0.81	-0.008	0.95	-0.036	0.75	-0.025	0.83
*K*_*i*_								
NAWM	0.019	0.87	0.132	0.25	-0.050	0.67	-0.020	0.86
WMH	0.097	0.40	0.172	0.14	0.010	0.93	0.081	0.49
CGM	0.039	0.73	0.091	0.43	0.014	0.91	0.001	0.99

*Overall* overall cognitive function, *Executive* executive function, *Speed* information processing speed, *v*_L_ leakage volume, *K*_i_ leakage rate, *NAWM* normal appearing white matter, *WMH* white matter hyperintensities, *CGM* cortical grey matter

β values represent standardized regression coefficients

**Table 4:** Association between cognition and *v*_L_ and *K*_i_, for NAWM, WMH and CGM, in healthy controls, uncorrected

	Overall		Executive		Speed		Memory	
	β	*p*-value	β	*p*-value	β	*p*-value	β	*p*-value
*v*_L_								
NAWM	0.000	1	0.096	0.56	0.021	0.90	-0.108	0.51
WMH	-0.094	0.57	0.020	0.91	-0.065	0.69	-0.192	0.24
CGM	-0.166	0.31	-0.67	0.68	-0.136	0.41	-0.226	0.17
*K*_*i*_								
NAWM	-0.237	0.15	**-0.326**	0.04*	**-0.339**	0.04*	0.029	0.86
WMH	-0.304	0.06	**-0.366**	**0.02**	**-0.340**	**0.03**	-0.105	0.52
CGM	0.204	0.21	0.051	0.31	0.143	0.39	**0.333**	**0.04**

*Overall* overall cognitive function, *Executive* executive function, *Speed* information processing speed, *vL* leakage volume, *Ki* leakage rate, *NAWM* normal appearing white matter, *WMH* white matter hyperintensities, *CGM* cortical grey matter.

βvalues represent standardized regression coefficients.

* p < 0.05 after correcting for age, sex, educational level and Hamilton Anxiety and Depression Score.

## Discussion

In this study on patients with clinically overt cSVD and age- and sex- matched healthy controls, we examined the association between BBB leakage and cognitive performance as well as WMH volume. We showed that in cSVD patients, larger WMH volume was associated with larger (fractional) leakage volume and lower leakage rate in the WMH. Furthermore, we found that in healthy controls, higher leakage rate in the NAWM is associated with lower cognitive scores on executive function and information processing speed, whereas no relation was found with any of the cognitive domains in cSVD patients.

BBB leakage is considered to play a pivotal role in the pathophysiological mechanisms of cSVD (Wardlaw et al. 2013a). Our results support this hypothesis by showing that with a larger WMH volume (which can be considered a marker for disease severity), more leakage in terms of a larger fractional leaking volume was present within these WMH. One could argue that more BBB leakage in the WMH is simply the result of tissue damage, instead of a preceding step in the occurrence of WMH. Longitudinal studies are needed to unravel a possible cause-consequence relationship between BBB leakage and WMH volume.

The finding that lower leakage rate in WMH was associated with a larger WMH volume was unexpected. This observation might be related to a decreased perfusion in the WMH (Brickman et al. 2009). However, further studies into this subject are needed.

It has been shown that in cSVD patients, the NAWM shows microstructural and BBB changes, suggestive for early pathological changes (van Norden et al. 2012; Zhang et al. 2017). In the current study, we did not find an association between BBB leakage in the NAWM and the severity of cSVD in terms of WMH volume. It is possible that leakage in the NAWM precedes development of visible WMH and therefore, no association can be detected between NAWM leakage and the current volume of WMH. Longitudinal studies can provide adequate insights in the temporal relationship between BBB leakage in the NAWM and occurrence of WMH.

Other studies also investigated the association between BBB leakage in the white matter and WMH severity. A DCE-MRI study in patients with lacunar stroke used contrast enhancement as a semi-quantitative measure for BBB leakage and showed that contrast-enhancement in the NAWM but not in the WMH was correlated with the visual scoring grade of WMH (Topakian et al. 2010). Yet, another study in patients with VCI did not find an association between WMH load and BBB leakage in the white matter, but they did not distinguish between NAWM and WMH (Huisa et al. 2015). A recent study in stroke patients showed that BBB leakage in both NAWM and WMH increased with WMH load but only in young patients with cSVD (Munoz Maniega et al. 2017). These inconsistent results reflect our still incomplete understanding of the mechanism of cSVD and the non-uniform state of development of BBB leakage measurement methods.

We found no significant association between BBB leakage and WMH volume in healthy controls, which differs from earlier findings (Farrall and Wardlaw 2009). This may be due to the low presence of WMH in the control group. It may also be due to the differences in methodology: previous studies compared BBB leakage between groups with limited and extended WMH whilst our study examined the linear relationship between BBB leakage and WMH volume. Moreover, previous studies used biochemical methods or imaging techniques that differ from our DCE-MRI measurement.

We found no association between BBB leakage and cognition in cSVD patients. This is consistent with earlier cross-sectional findings (Wardlaw et al. 2017). We did find an association between cognitive function and BBB leakage in healthy controls. This may indicate that BBB leakage is related to cognitive dysfunction in normal aging but no longer has a determining role in the cognitive functioning in cSVD due to dominant roles of more severe, vascular and parenchymal changes seen in cSVD. An association, if present, may be cofounded by other mechanisms such as lacunar infarcts and changes in cerebral perfusion and vasoreactivity.

This study has several strengths. Firstly, we included a well-represented spectrum of patients with clinically overt cSVD, with different stages of disease severity, and also examined age- and sex- matched healthy controls. Although the patients had different clinical features, the underlying small vessel pathology is presumed to be the same. We used clear in- and exclusion criteria and tried to include a population with cSVD pathology with as little concurrent diseases that may influence the BBB permeability as possible. Secondly, we have examined both leakage volume and leakage rate. Although leakage volume is a relatively new measure, we previously found that leakage volume is a measure that differentiates cSVD patients from age and gender matched controls, suggesting that leakage volume may be an equally important or perhaps even a more sensitive measure for BBB leakage than leakage rate (Zhang et al. 2017). Moreover, recent studies have shown that the method we used is suitable for determining subtle leakage, and our leakage rate values are comparable with leakage rate values found in earlier studies (Barnes et al. 2016; Montagne et al. 2015). Thirdly, by using quantitative measures for BBB leakage instead of earlier used semi-quantitative measures such as contrast enhancement, we could separate the filling of blood vessels with contrast material from leakage and examine the direct relationship between BBB leakage and WMH volume and cognition. This enables future analysis and comparison with other quantitative data, and with longitudinal data.

It is plausible that BBB leakage and cognitive function or WMH volume are associated in a time-related manner. The cross-sectional nature of our study may therefore be insufficient to detect such associations and may therefore be considered a limitation. Another limitation may be our relative small size of our healthy control group. However, despite this, we found significant associations between BBB leakage and cognition in this group.

In this study, we have presented quantitative data on the relation between BBB leakage and cognitive function and WMH volume in cSVD and normal aging. A larger WMH volume is associated with a larger leakage volume within these WMH in cSVD. We could not demonstrate a relation between BBB leakage and cognitive function in cSVD but we did find such a relationship in normal aging. Larger and longitudinal studies are desirable to gain further insight in the complex relationship between BBB leakage and the development of WMH and onset of cognitive dysfunction in cSVD and aging.

These funds did not have a role in the study process and did not contribute to the article.

## Electronic supplementary material

Below is the link to the electronic supplementary material.

Supplementary material 1 (DOCX 83 KB)
