# Supplementary material for: Blood–brain barrier leakage in relation to white matter hyperintensity volume and cognition in small vessel disease and normal aging
Source: Brain Imaging Behav. 2018 Mar 23;13(2):389–95. doi: 10.1007/s11682-018-9855-7 (PMC6486901; doi:10.1007/s11682-018-9855-7)
Supplement: Supplementary file 1 — Supplementary material 1 (DOCX 83 KB) [file 11682_2018_9855_MOESM1_ESM.docx]

**Supplemental data**

**Journal**: Brain Imaging and Behavior

Blood-brain barrier leakage in relation to white matter hyperintensity volume and cognition in small vessel disease and normal ageing

C. Eleana Zhang^1,3,4^, MD; Sau May Wong^2,4^, MSc; Renske Uiterwijk^1,4^, MSc; Walter H. Backes, PhD^2,4^; Jacobus F.A. Jansen, PhD^2,4^; Cécile R.L.P.N. Jeukens, PhD^2^; Robert J. van Oostenbrugge, MD, PhD^1,3,4^; Julie Staals, MD, PhD^1,3^;

^1^ *Department of Neurology, Maastricht University Medical Centre, The Netherlands*

*^2^ Department of Radiology & Nuclear Medicine, Maastricht University Medical Centre, The Netherlands*

*^3^ Cardiovascular Research Institute Maastricht (CARIM), The Netherlands*

*^4^ School for Mental Health and Neuroscience (MHeNs), The Netherlands*

**Corresponding Author:**

Eleana Zhang; Maastricht UMC, P. Debyelaan 25, 6202 AZ Maastricht, the Netherlands; Telephone: +31 433872907; Fax: +31 433877055; E-mail: [eleana.zhang@mumc.nl](mailto:eleana.zhang@mumc.nl)

ORCID ID: 0000-0002-8635-834X

Table A.1. Neuropsychological test scores in cSVD patients and healthy controls

|  | cSVD  Mean Score (SD) | Controls  Mean Score (SD) | *p-*value |
| --- | --- | --- | --- |
| Rey Auditory Verbal Learning Test |  |  |  |
| Inmediate recall | 32.0 (10.3) | 36.1 (7.7) | 0.03 |
| Delayed recall | 5.3 (3.5) | 6.6 (2.5) | 0.04 |
| Delayed recognition | 11.4 (3.2) | 12.6 (2.0) | 0.03 |
| Stroop Colour-Word Test Part 1# | 58.5 (15.3) | 50.9 (10.0) | 0.006 |
| Stroop Colour-Word Test Part 2# | 77.1 (20.5) | 65.7 (13.5) | 0.002 |
| Stroop Colour-Word Test Part 3# | 156.6 (64.3) | 119.5 (48.8) | 0.002 |
| Trail Making Test A# | 66.8 (34.1) | 44.2 (15.3) | <0.001 |
| Trail Making Test B# | 181.0 (121.7) | 106.4 (48.4) | <0.001 |
| Category Fluency | 30.2 (10.5) | 39.0 (12.3) | <0.001 |
| Letter Fluency | 24.6 (12.4) | 28.7(10.5) | 0.09 |
| Symbol Substitution - Coding | 42.2 (18.3) | 53.5 (18.3) | 0.002 |
| Digit Span Forward | 7.4 (2.0) | 7.8 (1.6) | 0.24 |
| Digit Span Backward | 5.3 (1.7) | 5.3 (1.5) | 0.21 |
| Letter Number Sequencing | 6.7 (3.3) | 7.6 (2.9) | 0.16 |

# Test score unit is in seconds
